# Neutrophil-to-lymphocyte and platelet-to-lymphocyte ratios as predictive and prognostic markers in patients with locally advanced rectal cancer treated with neoadjuvant chemoradiation

**DOI:** 10.1186/s12885-019-5892-x

**Published:** 2019-07-05

**Authors:** Shaan Dudani, Horia Marginean, Patricia A. Tang, Jose G. Monzon, Soundouss Raissouni, Timothy R. Asmis, Rachel A. Goodwin, Joanna Gotfrit, Winson Y. Cheung, Michael M. Vickers

**Affiliations:** 1 The Ottawa Hospital Cancer Centre/University of Ottawa, Ottawa, Ontario Canada; 2 Alberta Health Services/University of Calgary, Calgary, Alberta Canada; 3 Alberta Health Services/University of Calgary, Medicine Hat, Alberta Canada; 4 British Columbia Cancer Agency, Vancouver, British Columbia Canada; 5 Present address: Division of Medical Oncology, Tom Baker Cancer Centre, University of Calgary, 1331 29 St NW, Calgary, AB T2N 4N2 Canada

**Keywords:** Biomarkers, Survival, Pathologic complete response, Inflammatory response, Personalized medicine

## Abstract

**Background:**

A standard therapy for locally advanced rectal cancer (LARC) includes fluoropyrimidine (FP)-based neoadjuvant chemoradiation (nCRT). Previous studies have inconsistently demonstrated that baseline neutrophil- and platelet-to-lymphocyte ratios (NLR and PLR) are predictive of response to nCRT or prognostic of outcomes in LARC.

**Methods:**

We reviewed patients with LARC undergoing nCRT followed by surgery from 2005 to 2013 across 8 Canadian cancer centres. Outcome measures of interest were pathological complete response (pCR), disease-free survival (DFS) and overall survival (OS). Logistic regression and Cox proportional hazard models were used to assess for associations between baseline hematologic variables and outcomes.

**Results:**

Of 1527 identified patients, 1237 (81%) were included in the DFS/OS analysis. Median age was 62 (range 23–88), 69% were male, and 80% had performance status (PS) 0–1. Twenty-six percent had elevated NLR (≥ 4), and 66% had elevated PLR (≥ 150). Ninety-seven percent of patients received FP-based nCRT, with 96% receiving ≥44 Gy. 81% completed neoadjuvant chemotherapy and 95% completed neoadjuvant radiotherapy, with a pCR rate of 18%. After a median follow-up time of 71 months, 8% developed local recurrence, 22% developed distant recurrence and 24% died. 5-year DFS and OS were 69% (95% CI 66–72%) and 79% (95% CI 77–82%), respectively. In multivariate analyses, elevated baseline NLR and PLR were neither prognostic for DFS and OS nor predictive of pCR.

**Conclusions:**

NLR and PLR were not found to be independently prognostic for DFS or OS and did not predict for pCR in patients with LARC undergoing nCRT followed by surgery.

## Background

A standard of care treatment for locally advanced rectal cancer (LARC) includes fluoropyrimidine-based concurrent neoadjuvant chemoradiotherapy (nCRT) followed by total mesorectal excision (TME) [1, 2]. As compared to post-operative chemoradiation, use of nCRT in LARC is associated with improved rates of local control, tumour downstaging and sphincter-sparing surgery, as well as an improved toxicity profile [3]. However, response to nCRT varies widely between patients. Although roughly three-quarters of patients demonstrate evidence of response on postoperative histopathologic evaluation, with a proportion (usually < 25%) demonstrating pathologic complete response (pCR), up to one quarter of patients exhibit resistance to nCRT, displaying either minimal regression or complete lack of response [4–6]. Patients with radiographic and/or pathologic evidence of response to neoadjuvant therapy have been demonstrated to have improved long-term outcomes, including disease-free survival (DFS) and overall survival (OS) [5–9].

The mechanism(s) underlying the observed heterogeneity of tumour sensitivity to nCRT are not well understood, and currently there are no effective pre-operative models or biomarkers to predict response to nCRT. The ability to predict response and prognosis in patients undergoing nCRT for LARC could allow for cancer-directed treatments to be delivered in a more individualized manner. For example, patients predicted to have exquisite sensitivity to nCRT may be candidates for emerging “watch and wait”, organ-preserving strategies that may spare patients from the significant morbidity associated with rectal surgery [10–13]. Alternatively, patients with tumours predicted to be resistant to nCRT may be candidates for alternate neoadjuvant approaches (such as ‘total neoadjuvant therapy’, which incorporates preoperative chemotherapy in addition to nCRT [14]) or be considered for treatment with upfront surgery.

A range of clinical, radiologic, serologic, histopathologic and genetic factors have been studied as potential predictors of response to nCRT in LARC [8, 15–18]. Among these, the neutrophil-to-lymphocyte ratio (NLR) and platelet-to-lymphocyte ratio (PLR) are two readily-available serologic biomarkers which are felt to be surrogates for the degree of systemic inflammation and have been studied as prognostic markers in a range of malignancies [19, 20]. Previous studies have yielded conflicting results as to their prognostic/predictive potential in rectal cancer [19–28], though few studies have compared the roles of NLR and PLR in the same cohort. We conducted a multi-institutional review to assess the ability of NLR and PLR to predict prognosis and likelihood of response in patients with LARC treated with nCRT.

## Methods

### Study design and patient selection

Patients were identified and data were extracted from the Canadian Health Outcomes Research Database (CHORD) Consortium’s Rectal Cancer Database, which is a national, multi-institutional registry of locally advanced rectal cancer patients who have undergone nCRT followed by curative intent-surgery from four academic (British Columbia Cancer Agency, Cross Cancer Institute, The Ottawa Hospital Cancer Centre, Tom Baker Cancer Centre) and four community (Central Alberta Cancer Centre, Grand Prairie Cancer Centre, Jack Ady Cancer Centre, Margery E. Yuill Cancer Centre) cancer centres in Canada.

Patients were eligible for inclusion if they had: pathologically-confirmed rectal adenocarcinoma; clinical stage II or III disease as per the seventh edition of the American Joint Commission on Cancer staging system [29]; commenced long-course nCRT; underwent curative-intent surgery; baseline hematologic markers available (within 4 weeks prior and 2 weeks after initiating nCRT); documented absence of metastases (confirmed by CT or MRI of the abdomen and either chest radiograph or CT thorax). Patients were excluded if they had prior treatments for rectal cancer, evidence of metastatic disease, did not receive surgery, or received neoadjuvant radiation alone.

### Baseline hematologic variables

NLR was calculated by dividing the absolute neutrophil count by the absolute lymphocyte count. PLR was calculated by dividing the platelet count by the absolute lymphocyte count. NLR was defined as elevated if ≥4 and PLR was defined as elevated if ≥150. These cut-points were chosen based on systematic reviews of prior studies that used these thresholds and established them to be potentially predictive/prognostic [19, 20]. Restricted cubic spline analysis was also used to assess non-linear associations between NLR/PLR levels and survival endpoints, to determine if NLR ≥ 4 and PLR ≥ 150 were appropriate cut-points [30].

### Statistical analysis

We summarized patients’ demographics and baseline characteristics using descriptive statistics. When missing data were encountered, continuous variables were categorized and missing data were coded as not available (NA). Outcome measures of interest included DFS, OS and pCR. DFS was defined as time from diagnosis to first event (local recurrence, distant recurrence, or death from any cause) and censored at the date of last follow-up. OS was defined as the time from diagnosis to death from any cause and censored at the date of last follow-up. pCR was defined as the absence of any residual tumour cells on post-operative histologic evaluation of the rectal surgical specimen.

DFS and OS were evaluated using the Kaplan-Meier method. Uni- and multi-variable Cox regressions were conducted to determine the prognostic value of NLR and PLR on outcomes (DFS, OS) after adjustment for confounders. The assumptions of proportional hazards were checked for all final models. The covariate ‘province’ did not meet the assumption of the Cox regression. ‘Adjuvant chemotherapy’ was associated with lower risk of death but also did not meet the assumption of proportional hazards required for valid inference when using Cox proportional hazards. As a result, multivariable survival analysis was performed by stratifying on the two variables ‘province’ and ‘adjuvant chemotherapy’. A logistic regression model was also constructed to explore NLR and PLR as independent predictors of pCR.

The covariates were screened using univariate analyses and dropped from further inclusion in multivariate models if their crude association’s *p*-value with the outcomes was > 0.2. The remaining variables and their interactions were combined in a multivariate model. The interaction terms were assessed first for elimination from the model using a likelihood ratio test, significant at the 10% level (*p* = 0.1). The covariates were then assessed utilizing 2 methods: i) significance at the 10% level (p = 0.1) and, ii) the 10% change-in-estimate approach, where a variable was kept in the multivariate model if significant and its exclusion resulted in a substantial (> 10%) change to the survival coefficient estimate. Factors significant at the 0.05 level were retained in the multivariate model. Akaike’s Information Criterion and Bayesian Information Criterion were used to select the best models for NLR and PLR effect on outcomes.

Estimates (hazard ratios, odds ratios) are presented with 95% confidence intervals (95% CIs). We considered a *p*-value of 0.05 to be significant. All statistical analyses were performed using Stata® software, version 13.1 (Stata Corp LP, College Station, TX).

## Results

### Patient and tumour characteristics

**Fig. 1:**
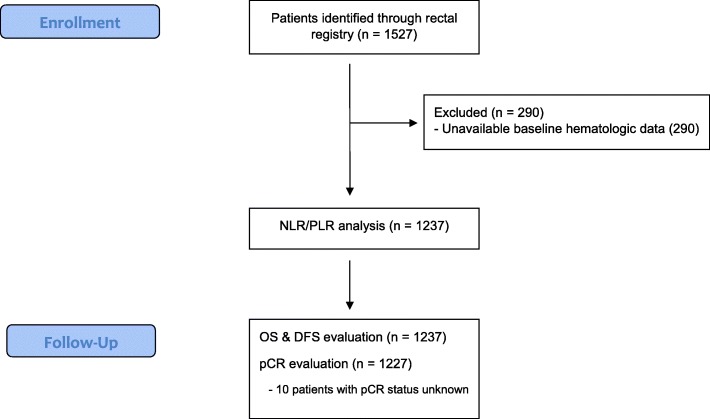
Patient Flow Diagram

Of 1527 identified patients, 1237 (81%) met eligibility criteria and were included for analysis (Fig. 1). All ineligible patients were excluded due to having unavailable baseline hematologic data. Patient demographics and tumour characteristics are summarized in Table 1.

**Table 1:** Baseline Patient and Tumour Characteristics

Variable		Total (*n* = 1237)	NLR		*p*-val	PLR		*p-*val
			< 4 (*n* = 912, 74%)	≥ 4 (*n* = 325, 26%)		< 150 (*n* = 418, 34%)	≥ 150 (*n* = 819, 66%)	
Province, n (%)	Alberta	595 (48)	435 (48)	160 (49)	*NS*	226 (54)	369 (45)	*< 0.01*
	British Columbia	255 (21)	186 (20)	69 (21)		69 (17)	186 (23)	
	Ontario	387 (31)	291 (32)	96 (30)		123 (29)	264 (32)	
Age, years	Median (range)	62 (23–88)	61 (23–88)	64 (24–87)	*< 0.01*	62 (23–85)	62 (23–88)	*NS*
	≥65, n (%)	482 (39)	334 (37)	148 (46)	*< 0.01*	154 (37)	328 (40)	*NS*
Male, n (%)		858 (69)	632 (69)	226 (70)	*NS*	302 (72)	556 (68)	*NS*
BMI, kg/m^2^ | *n* = 1130	≥ 25, n (%)	729 (59)	553 (61)	176 (54)	*NS*	272 (65)	457 (56)	*< 0.01*
ECOG PS, n (%)	0	524 (42)	398 (44)	126 (39)	*< 0.01*	188 (45)	336 (41)	*NS*
	1	466 (38)	344 (38)	122 (38)		160 (38)	306 (37)	
	2+	62 (5)	32 (4)	30 (9)		13 (3)	49 (6)	
	Unknown	185 (15)	138 (15)	47 (14)		57 (14)	128 (16)	
Distance from anal verge, n (%) | *n* = 1166	Median (range)	6 (0–30)	6 (0–30)	6 (0–25)	*NS*	6 (0–20)	6 (0–30)	*NS*
	< 5 cm	415 (34)	306 (33)	109 (34)	*NS*	137 (33)	278 (34)	*NS*
	5–10 cm	507 (41)	381 (42)	126 (39)		178 (43)	329 (40)	
	> 10 cm	244 (20)	181 (20)	63 (19)		84 (20)	160 (20)	
	Unknown	71 (6)	44 (5)	27 (8)		19 (4)	52 (6)	
Pre-treatment CEA, n (%) | *n* = 1093	Median (range)	4 (0–1133)	3 (0–857)	4 (0–1133)	*NS*	3 (0–468)	4 (0–1133)	*NS*
	< 5 ng/mL	667 (54)	510 (56)	157 (48)	*NS*	230 (55)	437 (53)	*NS*
	≥ 5 ng/mL	426 (34)	301 (33)	125 (39)		142 (34)	284 (35)	
	Unknown	144 (12)	101 (11)	43 (13)		46 (11)	98 (12)	
Clinical stage, n (%)	II	341 (28)	253 (28)	88 (27)	*NS*	119 (28)	222 (27)	*NS*
	III	880 (71)	644 (71)	236 (73)		291 (70)	589 (72)	
	Unknown	16 (1)	15 (2)	1 (0)		8 (2)	8 (1)	
Hemoglobin (g/dl)	Median (range)	136 (68–183)	137 (68–183)	131 (68–178)	*< 0.01*	140 (93–183)	133 (68–178)	*< 0.01*

*BMI* Body Mass Index, *ECOG PS* Eastern Cooperative Oncology Group Performance Status, *CEA* Carcinoembryonic Antigen, *NLR* Neutrophil-to-lymphocyte ratio, *NS* Non-significant, *PLR* Platelet-to-lymphocyte ratio

The median age was 62 (range 23–88), with 69% male and 80% Eastern Cooperative Oncology Group (ECOG) performance status 0–1. Twenty-six percent had NLR ≥ 4 and 66% had PLR ≥ 150. Median pre-treatment carcinoembryonic antigen (CEA) level was 4 ng/ml. Clinical stage II and III disease was noted in 28 and 71% of patients, respectively. Patients were followed for a median of 71 months.

### Treatments

Median radiation dose received was 50 Gy (range 20–80), with 96% receiving ≥44 Gy. Ninety-seven percent of patients received fluoropyrimidine-based neoadjuvant chemotherapy (35% capecitabine, 62% 5-fluorouracil), while 1% received neoadjuvant raltitrexed (2% not reported). Neoadjuvant chemotherapy and radiotherapy were completed as planned in 81 and 95% of patients, respectively. Fifty-three percent of patients underwent low anterior resection, 43% underwent abdominoperineal resection, and 3% underwent pelvic exenteration. Circumferential resection margin was > 1 mm (uninvolved) in 86%, ⩽ 1 mm (involved) in 8%, and unknown in 6%. The majority (80%) underwent TME within 6–12 weeks of completion of nCRT. Adjuvant chemotherapy was used in 73% of patients, with 28% of the total group receiving oxaliplatin-based adjuvant chemotherapy. Treatment details are summarized in Table 2.

**Table 2:** Treatment details

Variable		Total (*N* = 1237)	NLR		*p*-val	PLR		*p*-val
			< 4 (*n* = 912, 74%)	≥ 4 (*n* = 325, 26%)		< 150 (*n* = 418, 34%)	≥ 150 (*n* = 819, 66%)	
Neoadjuvant chemotherapy, n (%)	5-fluorouracil	764 (62)	558 (61)	206 (63)	*NS*	256 (61)	508 (62)	*NS*
	Capecitabine	430 (35)	322 (35)	108 (33)		149 (36)	281 (34)	
	Raltitrexed	15 (1)	14 (1)	1 (0)		5 (1)	10 (1)	
	Unknown	28 (2)	18 (2)	10 (3)		8 (2)	20 (3)	
Radiotherapy dose (Gy), n (%)	Median (range)	50 (20–80)	50 (20–74)	50 (29–80)	*NS*	50 (20–74)	60 (22–80)	*NS*
	< 44	36 (3)	32 (3)	4 (1)	*0.02*	15 (4)	21 (3)	*NS*
	44–46	225 (18)	156 (17)	69 (21)		65 (15)	160 (19)	
	≥ 46	966 (78)	719 (79)	247 (76)		333 (80)	633 (77)	
	Unknown	10 (1)	5 (1)	5 (2)		5 (1)	5 (1)	
Time from nCRT completion to TME	< 6 weeks	190 (15)	139 (15)	51 (16)	*NS*	62 (15)	128 (16)	*NS*
	6–12 weeks	988 (80)	725 (80)	263 (81)		335 (80)	653 (80)	
	> 12 weeks	57 (5)	46 (5)	11 (3)		20 (5)	37 (4)	
	Unknown	2 (0)	2 (0)	0 (0)		1 (0)	1 (0)	
Adjuvant chemotherapy, n (%)	5-fluorouracil	214 (17)	159 (17)	55 (17)	*NS*	78 (19)	136 (17)	*NS*
	Capecitabine	336 (27)	249 (27)	87 (27)		99 (24)	237 (29)	
	5-fluorouracil / oxaliplatin	275 (22)	199 (22)	76 (23)		96 (23)	179 (22)	
	Capecitabine / oxaliplatin	64 (5)	50 (5)	14 (4)		32 (8)	32 (4)	
	Other	10 (1)	8 (1)	2 (1)		4 (0)	6 (1)	
	No adjuvant chemotherapy	338 (27)	247 (27)	91 (28)		109 (26)	229 (28)	
Completed treatment as planned, n (%)	Neoadjuvant radiotherapy	1171 (95)	859 (94)	312 (96)	*NS*	394 (94)	777 (95)	*NS*
	Neoadjuvant chemotherapy	1001 (81)	734 (80)	267 (82)	*NS*	333 (80)	668 (82)	*NS*
Type of surgery, n (%)	Low anterior resection	657 (53)	490 (54)	167 (51)	*NS*	231 (55)	426 (52)	*NS*
	Abdominoperineal resection	535 (43)	397 (43)	138 (42)		175 (42)	360 (43)	
	Pelvic Exenteration	32 (3)	14 (2)	18 (6)		9 (2)	23 (3)	
	Unknown	13 (1)	11 (1)	2 (1)		3 (1)	10 (1)	
Total mesorectal excision, n (%)	Yes	1066 (86)	783 (86)	283 (87)	*NS*	372 (89)	694 (84)	*NS*
	No	20 (2)	12 (1)	8 (2)		7 (2)	13 (2)	
	Unknown	151 (12)	117 (13)	34 (11)		39 (9)	112 (14)	
Circumferential resection margin, n (%)	> 1 mm (uninvolved)	1061 (86)	782 (86)	279 (86)	*NS*	358 (86)	703 (85)	*0.03*
	⩽ 1 mm (involved)	103 (8)	72 (8)	31 (10)		27 (6)	76 (9)	
	Unknown	73 (6)	58 (6)	15 (5)		33 (8)	40 (5)	

*nCRT* Neoadjuvant chemoradiotherapy, *NLR* Neutrophil-to-lymphocyte ratio, *NS* Non-significant, *PLR* Platelet-to-lymphocyte ratio, *TME* Total Mesorectal Excision

### Outcomes

After a median follow-up time of 71 months, 8% developed local recurrence (LR), 22% developed distant recurrence (DR) and 24% had died. Median DFS was 132 months (95% CI 127 months – not reached), while median OS was not reached. 5-year DFS and OS rates were 69% (95% CI 66–72%) and 79% (95% CI 77–82%), respectively. pCR rate was 18%.

### Univariate and multivariate analyses

Factors included in univariate analyses were: age, sex, province, year of diagnosis (pre vs. post 2010), body mass index (BMI), statin use, ECOG performance status, pre-treatment CEA, clinical stage, distance from anal verge, RT dose (< 44 Gy vs ≥44 Gy), type of neoadjuvant chemotherapy (capecitabine vs. 5-fluorouracil vs. other), adjuvant chemotherapy use, baseline hemoglobin, NLR and PLR. Those significant in univariate analysis are listed in Table 3.

**Table 3:** Univariate Analyses

Variable	DFS [HR (95% CI)]	OS [HR (95% CI)]	pCR [OR (95% CI)]
NLR			
< 4	ref	^a^	^a^
≥ 4	1.24 (1.00–1.53)		
Age at diagnosis			
< 65	ref	ref	ref
≥ 65	1.34 (1.11–1.63)	1.77 (1.40–2.22)	0.72 (0.53–0.98(
ECOG PS			
0	ref	ref	ref
1	1.53 (1.22–1.93)	1.81 (1.36–2.39)	0.72 (0.52–1.00)
2+	2.06 (1.37–3.10)	3.37 (2.17–5.25)	0.19 (0.06–0.63)
Unknown	1.75 (1.33–2.31	2.04 (1.47–2.83)	0.79 (0.51–1.23)
Distance from anal verge			
< 5 cm	ref	ref	^a^
5–10 cm	0.92 (0.74–1.16)	0.90 (0.69–1.18)	
> 10 cm	0.79 (0.59–1.05)	0.72 (0.51–1.02)	
Unknown	1.49 (1.02–2.15)	1.52 (1.00–2.33)	
Pre-treatment CEA (ng/ml)			
< 5	ref	ref	ref
≥ 5	1.71 (1.39–2.11)	1.83 (1.43–2.34)	0.37 (0.26–0.54)
Unknown	1.63 (1.21–2.19)	1.49 (1.04–2.13)	0.69 (0.43–1.12)
Clinical stage			
II	ref	ref	ref
III	1.21 (0.97–1.52)	1.07 (0.82–1.39)	0.66 (0.49–0.91)
Unknown	3.35 (1.75–6.43)	4.14 (2.07–8.26)	0.25 (0.03–1.92)
Hemoglobin^b^	0.99 (0.99–1.00)	0.99 (0.98–1.00)	1.01 (1.00–1.02)
Neoadjuvant chemotherapy			
5-fluorouracil	ref	ref	^a^
Capecitabine	1.30 (1.06–1.60)	1.27 (0.99–1.63)	
Raltitrexed	0.56 (0.18–1.75)	0.52 (0.13–2.08)	
Unknown	2.95 (1.82–4.76)	3.28 (1.90–5.65)	
Adjuvant chemotherapy^c^			
Not received	ref	ref	N/A
Received	0.63 (0.51–0.77)	0.45 (0.35–0.56)	N/A
Province^c^			
Alberta	ref	ref	ref
British Columbia	1.50 (1.19–1.90)	1.34 (1.02–1.75)	0.56 (0.37–0.86)
Ontario	0.65 (0.51–0.84)	0.47 (0.35–0.64)	0.83 (0.60–1.16)

*CEA* Carcinoembryonic antigen, *ECOG PS* Eastern Cooperative Oncology Group Performance Status, *HR* Hazard ratio, *OR* Odds Ratio

Non-significant in univariate analysis: PLR, sex, year of diagnosis, body mass index, statin use, radiation dose

^a^No statistically significant results

^b^Measured as continuous variable

^c^Non-proportional hazards

On multivariate analyses, independent predictors of shorter DFS were: elevated pre-treatment CEA, clinical stage III/unknown and lower hemoglobin levels, while independent predictors of shorter OS were elevated pre-treatment CEA, elevated PS and older age at diagnosis (≥ 65). Independent negative predictors of pCR were elevated pre-treatment CEA and clinical stage III. Elevated baseline NLR and PLR were not independently predictive of pCR, or prognostic for OS or DFS. Multivariate analyses are summarized in Table 4 (DFS), Table 5 (OS) and Table 6 (pCR).

**Table 4:** DFS Multivariate Analysis

Outcome	Hazard Ratio (95% CI)	*P*-value
NLR		
< 4	ref	0.14
≥ 4	1.19 (0.95–1.50)	
PLR		
< 150	ref	0.71
≥ 150	0.96 (0.76–1.21)	
Pre-treatment CEA		
< 5 ng/ml	ref	< 0.01
≥ 5 ng/ml	1.66 (1.34–2.05)	
Unknown	1.85 (1.37–2.51)	
Clinical stage		0.01
II	ref	
III	1.30 (1.02–1.65)	
Unknown	2.46 (1.24–4.90)	
Hemoglobin	0.99 (0.99–1.00)	0.02

*CEA* Carcinoembryonic antigen, *DFS* Disease-free survival, *NLR* Neutrophil-to-lymphocyte ratio, *PLR* Platelet-to-lymphocyte ratio

**Table 5:** OS Multivariate Analysis

Outcome	Hazard Ratio (95% CI)	*P*-value
NLR		
< 4	ref	0.99
≥ 4	1.00 (0.76–1.32)	
PLR		
< 150	ref	0.59
≥ 150	0.99 (0.76–1.29)	
Pre-treatment CEA		
< 5 ng/ml	ref	< 0.01
≥ 5 ng/ml	1.71 (1.33–2.20)	
Unknown	1.64 (1.13–2.38)	
ECOG Performance Status		< 0.01
0	ref	
1	1.43 (1.06–1.92)	
2+	2.24 (1.41–3.56)	
Unknown	1.29 (0.90–1.85)	
Age at diagnosis		
< 65	ref	< 0.01
≥ 65	1.50 (1.18–1.90)	

*CEA* Carcinoembryonic antigen, *ECOG* Eastern Cooperative Oncology Group, *NLR* Neutrophil-to-lymphocyte ratio, *OS* Overall survival, *PLR* Platelet-to-lymphocyte ratio

**Table 6:** pCR Multivariate Analysis

Outcome	Odds Ratio (95% CI)	*P*-value
NLR		
< 4	ref	0.16
≥ 4	0.76 (0.52–1.11)	
PLR		
< 150	ref	0.90
≥ 150	1.02 (0.73–1.42)	
Pre-treatment CEA		
< 5 ng/ml	ref	< 0.01
≥ 5 ng/ml	0.38 (0.26–0.55)	
Unknown	0.71 (0.44–1.14)	
Clinical stage		
II	ref	0.03
III	0.67 (0.49–0.92)	
Unknown	0.29 (0.04–2.29)	

*CEA* Carcinoembryonic antigen, *ECOG* Eastern Cooperative Oncology Group, *NLR* Neutrophil-to-lymphocyte ratio, *pCR* Pathologic complete response, *PLR* Platelet-to-lymphocyte ratio

## Discussion

Prognostication and treatment decisions in rectal cancer are primarily based on the anatomic extent of disease spread (i.e. staging), with few biologic tumour or host characteristics (biomarkers) employed to guide decision-making in this setting. However, there exists considerable heterogeneity in survival and response to treatment even among patients with similar stages of disease, suggesting that differences in host and/or tumour biology may play an important role in determining outcome in these patients. The elucidation of these heterogeneous biological factors may help to guide patient counseling and to personalize management decisions in patients with LARC. The latter may become an increasingly important issue as contemporary, alternative management strategies (including non-operative and ‘total’ neoadjuvant approaches) emerge as potential options for patients with LARC [1, 2, 12–14].

In recent years, the host immune and inflammatory response to malignancy have been demonstrated to be important factors in the development, progression, treatment and survival across a range of cancers [31, 32]. Indeed, tumour-promoting inflammation is a known hallmark of cancer [33]. Accordingly, an increased systemic inflammatory response as indicated by a range of surrogate biomarkers (e.g. elevated C-reactive protein, hypoalbuminemia, leukocytosis, thrombocytosis, etc.) – including NLR and PLR – have been shown to be associated with treatment response and outcome in a variety of malignancies, and several of these have been incorporated into prognostic scoring systems for various types of cancer [34–36]. In addition, some of these factors have also been incorporated into models intended to predict response to treatment [37, 38]. However, the precise mechanisms underlying these observations are complex and remain poorly understood [31].

NLR and PLR are two such biomarkers which are felt to be surrogates of the systemic inflammatory response and are potentially appealing as prognostic and predictive biomarker candidates because they are readily available and easily derived. However, the results of this study suggest that NLR and PLR are neither independently prognostic of outcome nor predictive of response to nCRT in LARC patients undergoing nCRT followed by curative-intent TME.

To our knowledge, this is the largest reported study of the prognostic and predictive impact of NLR and/or PLR in this patient population. Previous studies have reported conflicting results in this setting, with some demonstrating poorer prognosis with higher NLR and/or PLR [39, 40], while others did not observe any significant association [27]. Two prior meta-analyses investigating the prognostic role of NLR and PLR across a range of solid tumours demonstrated an association with adverse OS for both biomarkers in combined study populations of over 40,000 and 12,000 patients, respectively [19, 20].

However, these studies included a diverse range of tumour types and included patients with both non-metastatic and metastatic disease. The results of these meta-analyses may not be generalizable to patients with LARC as a greater association was noted in patients with metastatic disease. Of note, both meta-analyses also combined rectal cancer patients with colon cancer patients. There are some data to suggest that the prognostic and predictive capabilities of NLR and PLR differ between rectal and colon cancer as at least one study has demonstrated that NLR was significantly associated with adverse OS in colon, but not rectal, cancer [41]. In addition, it is important to note the potential impact of publication bias favouring positive results in studies of this nature.

With regard to their role in predicting response to neoadjuvant therapy, a recent systematic review and meta-analysis demonstrated an increased likelihood of pCR in patients with rectal cancer and low NLR receiving neoadjuvant chemotherapy +/− radiation (OR 2.01, 95% CI 1.14–3.55, *p* = 0.02) [42], which is inconsistent with our results. In addition, a recent study identified elevated PLR (> 133.4) to be a significant predictor of poor pathologic response in rectal cancer patients following nCRT [40]. The reasons for these discrepancies are unclear but may be related to differences in study design and analysis. For example, the systematic review included a total of seven studies pertaining to rectal cancer with various inclusion criteria, NLR/PLR cut-offs, and neoadjuvant treatment regimens (including non-radiotherapy-based treatment), while the latter study was not specific to pCR and did not prespecify cut-off values for the baseline hematologic variables (optimal values were derived from a receiver operating characteristic curve).

Several other prognostic features such as higher stage, poor performance status and elevated pre-treatment CEA emerged as significant prognostic and predictive factors in this cohort, which is consistent with prior studies [43, 44].

Strengths of our study include the relatively large sample size, long duration of follow up and multi-institutional cohort of patients from both academic and community cancer centres across Canada. In addition, the rates of pCR and 5-year OS and DFS rates observed compare favourably to several landmark trials of nCRT in LARC [3, 4, 45], which further supports the validity and generalizability of our results.

Limitations of our study include the retrospective design, which introduces the potential for unmeasured biases. In addition, approximately one fifth of screened patients were ineligible for inclusion due to missing data, and survival endpoints were not systematically recorded across all provinces, leading to higher proportions of censored in patients in some provinces (e.g. Ontario). Finally, the optimal cut-off values for NLR and PLR are not known and vary widely between studies [19, 20]. The cut-offs used in this study were chosen based on either the most commonly used or the median cut-off values identified in previously published systematic reviews [19, 20]. To ascertain that these values were appropriate cut-offs, we also performed cubic spline analyses for both NLR and PLR, which confirmed the suitability of these thresholds (data not shown). In addition, NLR has been shown to have a relatively consistent HR for OS across a range of cut-off values from 1.0–5.0 [20].

## Conclusions

In summary, we did not find any significant prognostic or predictive association for either NLR or PLR in LARC patients undergoing nCRT followed by TME. Ongoing efforts to identify prognostic and/or predictive biomarkers in LARC are warranted and may help to personalize management decisions in this patient population.

## Data Availability

The datasets used and/or analysed during the current study are available from the corresponding author on reasonable request.

## Abbreviations

BMI: Body mass index
CEA: Carcinoembryonic antigen
CHORD: Canadian Health Outcomes Research Database
CI: Confidence interval
DFS: Disease-free survival
DR: Distant recurrence
ECOG: Eastern Cooperative Oncology Group
FP: Fluoropyrimidine
LARC: Locally advanced rectal cancer
LR: Local recurrence
NA: Not available
nCRT: Neoadjuvant chemoradiotherapy
NLR: Neutrophil-to-lymphocyte ratio
OS: Overall survival
pCR: Pathological complete response
PLR: Platelet-to-lymphocyte ratio
PS: Performance status
RT: Radiotherapy
TME: Total mesorectal excision
